# Effects of *In Vivo* Gluten Challenge on PBMC Gene Expression Profiles in Diet Treated Celiac Disease

**DOI:** 10.3389/fimmu.2020.594243

**Published:** 2020-12-11

**Authors:** Dawit A. Yohannes, Andrea de Kauwe, Katri Kaukinen, Kalle Kurppa, Markku Mäki, Robert P. Anderson, Sten Linnarsson, Dario Greco, Päivi Saavalainen

**Affiliations:** ^1^Research Programs Unit, Translational Immunology, University of Helsinki, Helsinki, Finland; ^2^Department of Medical and Clinical Genetics, University of Helsinki, Helsinki, Finland; ^3^Department of Internal Medicine, Tampere University Hospital and Faculty of Medicine and Health Technology, Tampere University, Tampere, Finland; ^4^Department of Pediatrics, Tampere University Hospital, Tampere, Finland, Center for Child Health Research, Tampere University, Tampere, Finland; ^5^Department of Pediatrics, Seinäjoki University Consortium and Seinäjoki Central Hospital, Seinäjoki, Finland; ^6^Immunology Division, Walter and Eliza Hall Institute of Medical Research, Melbourne, VIC, Australia; ^7^Laboratory for Molecular Neurobiology, Department of Medical Biochemistry and Biophysics, Karolinska Institute, Stockholm, Sweden; ^8^Faculty of Medicine and Health Technology & BioMediTech Institute, Tampere University, Tampere, Finland; ^9^Institute of Biotechnology, University of Helsinki, Helsinki, Finland

**Keywords:** celiac disease, celiac disease gene expression analysis, celiac disease RNA sequencing, celiac disease transcriptomics, pathway analysis, gluten challenge

## Abstract

The pathological mechanisms that lead to the onset and reactivation of celiac disease (CD) remain largely unknown. While gluten free diet (GFD) improves the intestinal damage and associated clinical symptoms in majority of cases, it falls short of providing full recovery. Additionally, late or misdiagnosis is also common as CD presents with a wide range of symptoms. Clear understanding of CD pathogenesis is thus critical to address both diagnostic and treatment concerns. We aimed to study the molecular impact of short gluten exposure in GFD treated CD patients, as well as identify biological pathways that remain altered constitutively in CD regardless of treatment. Using RNAseq profiling of PBMC samples collected from treated CD patients and gluten challenged patient and healthy controls, we explored the peripheral transcriptome in CD patients following a short gluten exposure. Short gluten exposure of just three days was enough to alter the genome-wide PBMC transcriptome of patients. Pathway analysis revealed gluten-induced upregulation of mainly immune response related pathways, both innate and adaptive, in CD patients. We evaluated the perturbation of biological pathways in sample-specific manner. Compared to gluten exposed healthy controls, pathways related to tight junction, olfactory transduction, metabolism of unsaturated fatty acids (such as arachidonic acid), metabolism of amino acids (such as cysteine and glutamate), and microbial infection were constitutively altered in CD patients regardless of treatment, while GFD treatment appears to mostly normalize immune response pathways to “healthy” state. Upstream regulator prediction analysis using differentially expressed genes identified constitutively activated regulators relatively proximal to previously reported CD associated loci, particularly SMARCA4 on 19p13.2 and CSF2 on 5q31. We also found constitutively upregulated genes in CD that are in CD associated genetic loci such as MEF2BNB-MEF2B (BORCS8-MEF2B) on 19p13.11 and CSTB on 21q22.3. RNAseq revealed strong effects of short oral gluten challenge on whole PBMC fraction and constitutively altered pathways in CD PBMC suggesting important factors other than gluten in CD pathogenesis.

## Introduction

Celiac disease (CD) is an immune-mediated enteropathy associated with duodenal inflammation and villus atrophy. It may present with various gastrointestinal and extra-intestinal symptoms (1, 2). The worldwide prevalence of CD is estimated to be around 1% (3). The disease is driven by ingestion of gluten naturally present in wheat, barley, or rye. Gluten free diet (GFD) is currently the only available treatment, although in rare cases patients can be unresponsive and develop serious complications (4). CD has a strong genetic component with almost all patients carrying the class II HLA types HLA-DQ2 or DQ8. Other yet unidentified factors also contribute to the onset of CD since these haplotypes are common in the general population. Due to the diverse clinical presentation, there is a high risk for misdiagnosis or delayed diagnosis, exposing the patients for permanent complications (5). Deciphering the biological mechanisms that characterize CD could help to find better methods for early detection of the disease.

Multiple studies have assessed transcriptional changes associated with active CD intestinal mucosa (6–9). The overall picture has indicated gene expression changes associated with immune cell activation and proliferation, epithelial cell differentiation and an overall dysregulation of the proper balance between dying and dividing cells in the CD intestine, as well as possible involvement of metabolic pathways, microbial infection and intestinal barrier related pathways. Gene expression studies have also been used to show expression level differences in candidate genes obtained from CD associated genomic loci (10, 11). Most of the studies used intestinal biopsies, typically with samples collected from active CD patients or *in-vitro* stimulated cells, to assess expressional changes in CD (6, 7, 11, 12); in few cases blood samples were used to study specific cell types known to play a role in CD pathogenesis (10).

In this study, we were interested in exploring peripheral transcriptional changes induced by short gluten challenge in CD patients on a GFD, and persistently altered pathways that characterize CD pathogenesis. We evaluated gluten induced transcriptional changes in treated CD PBMC, following a short 3-day *in-vivo* gluten exposure, and further compared with gluten exposed HLA-DQ2+ healthy controls. The setup allowed us to also study persistently altered pathogenic pathways in treated patients in comparison to healthy controls carrying the at-risk genotype. While gluten induced systemic transcriptional changes mainly characterized by the activation of innate and adaptive immune response pathways, we found deregulation of pathways involving amino acid metabolism, tight junction, microbial infection, and olfactory transduction to be constitutively altered in CD despite GFD. We also detected previously unappreciated genes from known CD associated loci with constitutive involvement in CD pathogenesis.

## Materials and Methods

### Ethical Permission

The Ethical Committee of the Pirkanmaa Hospital District, Finland approved the study design and recruiting of subjects. Subjects gave written informed consent. Relevant guidelines and regulations were followed when performing the experiments.

### Study Subjects and Sample Collection

Twelve gluten free diet (GFD) treated adult celiac disease (CD) patients and three healthy controls who were on GFD for 4 weeks were selected for a case-control type analysis in this study from our big gluten challenge study cohort (unpublished), which is partly described in our recent published study (13). Eleven of the patients and all three healthy controls were positive for at least one copy of HLA-DQ2, and one patient positive for the other risk genotype HLA-DQ8. For the CD patients, presence of duodenal villous atrophy accompanied by elevated serum anti-tissue transglutaminase IgA at the time of diagnosis was confirmed. GFD adherence of all the CD patients was assessed to be strict before the challenge based on negative TGA and EMA antibodies, and an interview. Patients and controls underwent a 3-day oral wheat gluten challenge (4 slices, equivalent to approximately 10 g/d gluten). Peripheral blood mononuclear cells (PBMC) were separated from blood collected from the gluten unchallenged CD patient samples on day 0 (unchallenged CD), and the gluten challenged CD patient and control samples on day 6 (challenged CD and challenged HC respectively), as it is known that circulating IFNg+ cells following gluten exposure are highest on day 6 (14, 15). All samples were cryopreserved. Details of all subjects is provided on **Table S1**, and details of samples used for differential gene expression and pathway analysis is given on **Table 1**.

**Table 1:** Details of Celiac disease (CD) patients and healthy controls in the study.

	Unchallenged CD (CD day 0)	Challenged CD (CD day 6)	Challenged HC (HC day 6)	DE genes
**Samples (n)**	6	6	3	
**Sex (F,M)**	4,2	4,2	3,0	
**Mean Age (range)**	49 (22–66)	57 (37–68)	39 (30–45)	
**DQ2 + (%)**	5 (83)	6 (100)	3 (100)	
**GFD duration (range)**	6 (1–14) yrs	9 (1–16) yrs	4 wks	
**DE Analysis**	x	x		445
		x	x	166
	x		x	40

Number of differentially expressed genes detected for comparisons between sample groups is given.

### RNA Sequencing

Total RNA was extracted from the frozen PBMC stocks of the selected subjects using RNeasy Mini kit (Qiagen)­ for 12 patients (9 day-0 and 9 day-6 samples), and 3 healthy control samples (day-6 only), altogether 21 samples. We used the STRT (single-cell tagged reverse transcription) 5’-end RNA-sequencing method which is designed for capturing mRNAs from single cells, and is suitable for quick and cost-efficient gene expression profiling with very low starting RNA (16). Here we used STRT to prepare library from bulk PBMC samples with 4 ng RNA per sample. Two microliters of RNA lysis buffer was added to 1 μl of each RNA sample on a 96-well plate, incubated at 71°C for 3 min, and then cooled to 4°C for 10 min. A volume of 3.6 μl of the reverse transcription mix was added on the samples and incubated at 42°C for 90 min and subsequently at 70°C for 10 min. The acquired cDNA was amplified by PCR and quantified by Qubit Fluorometer (Life Technologies). Six nanograms of cDNA was simultaneously fragmented and barcoded by Tn5 tagmentation, isolated with beads, and quantified by KAPA Library Quant (Kapa Biosystems). Sequencing was performed on an Illumina HiSeq 2000 instrument generating 50-bp reads, including a 6-bp unique molecular identifier along with 8-bp index reads corresponding to the cell/well-specific barcode.

### Bioinformatics and Statistical Analysis

We obtained an average of 1.4 million valid STRT reads per sample after filtering out reads with low quality, lacking template switch GGG trinucleotide or with less than 25 nt insert size as per the standard STRT data analysis (16, 17). Redundant reads due to PCR duplication were corrected using the unique molecular identifiers (UMI). Remaining reads were aligned to the complete UCSC hg19 human genome using Bowtie (18). On average, 144670 UMIs (representing PCA bias corrected transcripts) covering 8,400 genes were obtained per sample.

Differential gene (DE) expression analysis was performed using edgeR (19) in R. Genes with adjusted p value of <0.05 with log2 fold change ≥1 or ≤-1 were considered significantly upregulated or downregulated, respectively. We performed three analyses comparing: 1) gluten challenged patients versus GFD treated patients (challenged CD versus unchallenged CD, 6 samples per group, 4 females and 2 males per group); 2) gluten challenged patients versus gluten challenged healthy controls (challenged CD versus challenged HC, 4 versus 3 samples, all females); and 3) GFD treated patients versus gluten challenged healthy controls (unchallenged CD versus challenged HC, 4 versus 3 samples, all females).

We used R for statistical and biological pathway analysis. R package fgsea (20) was used for gene set enrichment analysis (GSEA) (21), and Pathifier (22) was used for sample specific pathway deregulation score (PDS) estimation. GSEA and Pathifier are regarded as functional scoring pathway analysis tools that evaluate the relevance of gene sets representing biological pathways by utilizing information from all genes in the dataset instead of using only those that are called differentially expressed (23). This minimizes the effect of arbitrary cut-offs for the selection of genes and allows better detection of disease relevant pathways by utilizing the complete transcriptomic information in our datasets. GSEA gives enrichment scores for each pathway given the fold change (logFC) of all genes from a differential expression analysis. Pathifier on the other hand gives a sample-specific pathway deregulation score (PDS) for each pathway given total gene expression profile of a sample. The PDS is an estimate of how distant a particular pathway is from its “normal” state that is obtained from the control samples in the data (22), the healthy control day 6 samples were set to represent the “normal” state in our analysis. For both tools, we used the KEGG (Kyoto Encyclopedia of Genes and Genomes) (24) pathways curated and provided by Molecular Signatures Database (MSigDB) (25) as reference gene sets (the CP : KEGG manually curated pathway database was used which contains 186 gene sets). We have also separately examined the differentially expressed genes (DEGs) using Ingenuity pathway analysis (IPA: QIAGEN Inc., https://www.qiagenbioinformatics.com/products/ingenuity-pathway-analysis) for general pathway enrichment and upstream regulator prediction analysis. In addition to enrichment significance p-values, IPA also predicts activation and inhibition status for both canonical pathway enrichment and upstream regulator prediction tasks. It does this by matching the observed up and downregulation of pathway or regulator relevant genes among our DEGs to their expected direction of expression when participating in a pathway or when influenced by an upstream regulator, based on what is known from curated information collected in IPA knowledge base. It then provides a z-score statistic quantifying the effect with z-score >2 signifying significant activation or <-2 for inhibition (26). Online tools Clustvis (27) and InteractiVenn (28) were used to visualize some results.

## Results

### Differential Gene Expression in CD Following Short Gluten Exposure

**Figure 1:**
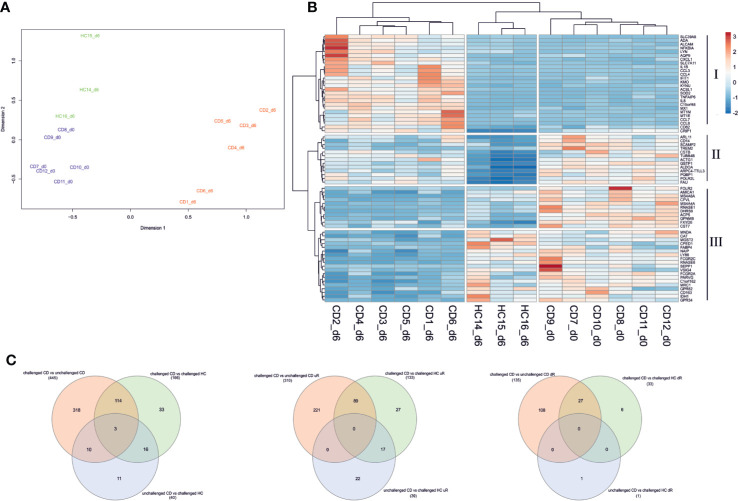
Differential gene expression in PBMC of CD patients following short gluten challenge. **(A)** Multidimensional scaling (MDS) plot of the total gene expression profile segregates pre-challenge patients (from day 0 shown in blue) from post-gluten challenge patients (day 6 samples in red). Gluten challenged healthy controls (in green) largely cluster with treated patients. **(B)** Top 20 differentially expressed genes from all three analysis are used to make a heatmap. The top 20 genes segregate samples by their health status. Gene clusters I and III appear to be altered genes following the 3-day gluten exposure, with I significantly upregulated and III downregulated in gluten exposed samples of day 6. Gene cluster II is downregulated in healthy controls following gluten exposure while remaining relatively highly expressed across patient samples from day 6 and particularly day 0. Rows are centered and unit variance scaling is applied to rows. Both genes and samples are clustered using hierarchical clustering with euclidean distance and ward linkage. **(C)** Venn diagram of DEGs from the three analyses is shown for all DEGs, differentially upregulated genes, and downregulated genes respectively. CD, celiac disease; HC, healthy control; d0, day0; d6, day6.

We first evaluated the total genome-wide gene expression profiles. Our samples generally showed distinct genome wide expression profiles in accordance with their health as well as gluten exposure status with some exceptions. Three day 6 samples of CD7, CD8, and CD11 appeared to cluster with gluten challenged healthy control samples, and three day 0 samples of CD3, CD4, and CD6 appeared to cluster with gluten challenged day 6 patient samples (**Figure S1**). Compared to other patients, patients CD7, CD8, and CD11 had a tendency towards higher proportions of no symptoms during the gluten challenge (2/3 vs 2/6; Chi-squared test, p = 0.3754) or none IFNg response upon gliadin stimulation (1/3 vs 0/6; Chi-squared test, p = 0.1597), perhaps the reason why they clustered with gluten challenged healthy controls. On the other hand, patients CD3, CD4, and CD6 might have had heightened immune response already before day 0 (**Table S1**), particularly CD3 was the only patient unable to complete the oral challenge due to strong clinical symptoms, perhaps explaining why their day 0 gene expression profiles appear closer to patient day 6 profiles. In view of such possible confounding factors, we decided to exclude these samples from downstream analyses. Overall, gluten challenged healthy controls generally clustered closer to GFD treated patients while gluten challenged patients formed a clearly distinct cluster, indicating that the short gluten exposure induced similarly altered genome-wide expression patterns among patients (**Figure 1A** and **Figure S1**).

Next, differential expression analysis of the selected samples with edgeR identified 445 differentially expressed genes (DEGs) between the gluten challenged CD and unchallenged CD patients, 166 DEGs between the gluten challenged CD and challenged HC subjects, and 40 DEGs between the unchallenged CD and challenged HC subjects (for a complete list of DEGs see **Supplementary File S1**). Our list of DEGs included three of the 79 CD candidate genes from the non-HLA CD risk loci (29), GLB1 (3p22.3, downregulated), CCR2 (3p21.31, downregulated), and TNFAIP3 (6q23.3, upregulated), all three from the challenged CD versus unchallenged CD analysis. We plotted the top 20 DEGs from the three analyses on a heatmap (**Figure 1B**). A clear segregation of the samples into two main branches of 1) gluten challenged patients, and 2) treated patients and the healthy controls (despite the gluten exposure) was observed. The gluten exposed healthy controls still formed a distinct group from the treated patients largely driven by downregulated genes in gene cluster II, which is conversely highly expressed in treated patients (**Figure 1B**). Related to these observations, our analyses also detected DEGs specific to each comparison, with for example DEGs from patient only comparisons (challenged CD to unchallenged CD) overlapping only partly with the DEGs detected from comparisons involving the healthy controls (**Figure 1C**). We sought to explore our data using pathway analysis to find molecular mechanisms that explain the gene expression profile of each sample group in our study.

### Pathway Analysis Using DEGs

Pathway analysis of the differentially expressed genes (DEGs) was performed for all three analyses using IPA. The number of significantly enriched IPA canonical pathways were 99 in the challenged CD versus unchallenged CD, 45 in the challenged CD versus challenged HC, and 3 in the unchallenged CD versus challenged HC analyses respectively (with adjusted p-values <0.05 or –log10 p-values >1.3). Of these, 42 were predicted to be activated and 3 inhibited in the challenged CD versus unchallenged CD, and 7 to be activated in the challenged CD versus challenged HC analyses (with z-score >2 or <-2). No activated or inhibited pathway was determined for the unchallenged CD versus challenged HC analysis. As was expected, the significantly enriched IPA pathway results were largely pro-inflammatory indicating mobilization of immune cells (enrichment of pathways such as Agranulocyte Adhesion and Diapedesis, Granulocyte Adhesion and Diapedesis, Neuroinflammation Signaling Pathway) as well as inflammatory signaling (enrichment of pathways such as IL-8 Signaling, NF-κB Signaling, IL-17 Signaling) (**Figure S2A**, for a complete list of enriched and activated/inhibited pathways see **Supplementary File S2**). Of particular interest, pathways that showed significant enrichment in treated (unchallenged CD) patients compared to controls were Fcγ Receptor-mediated Phagocytosis in Macrophages and Monocytes, Remodeling of Epithelial Adherens Junctions, and Actin Cytoskeleton Signaling, suggesting that treated patients still maintained abnormal activity of cytoskeletal and cell-to-cell adheren function as well as antibody driven phagocytic activity (**Figure S2A**). IPA also predicted the activation status of the significantly enriched pathways among the DEGs. Of the pathways that were predicted to be activated among DEGs induced by gluten exposure (challenged CD versus unchallenged CD, and challenged CD versus challenged HC analyses, **Figure S2B**), Role of IL-17F in Allergic Inflammatory Airway Diseases was interesting. IL-17F was recently shown to be increased in CD patient serum, and was found to correlate with the density of enteroviruses (EV)-positive cells in the lamina propria of CD patients (30). Its enrichment following gluten exposure and predicted activation could indicate its increased inflammatory activity in CD, especially in the presence of possible active or historic microbial infection. Retinoate biosynthesis, RHoGDI and Peroxisome proliferator-activated receptor (PPAR) signaling were predicted to be inhibited in challenged CD versus unchallenged CD DEGs, while liver X receptor/retinoid X receptor (LXR/RXR) Activation pathway was inhibited in both challenged CD versus unchallenged CD, and challenged CD versus challenged HC DEGs.

### Pathway Analysis Using All Genes

**Figure 2:**
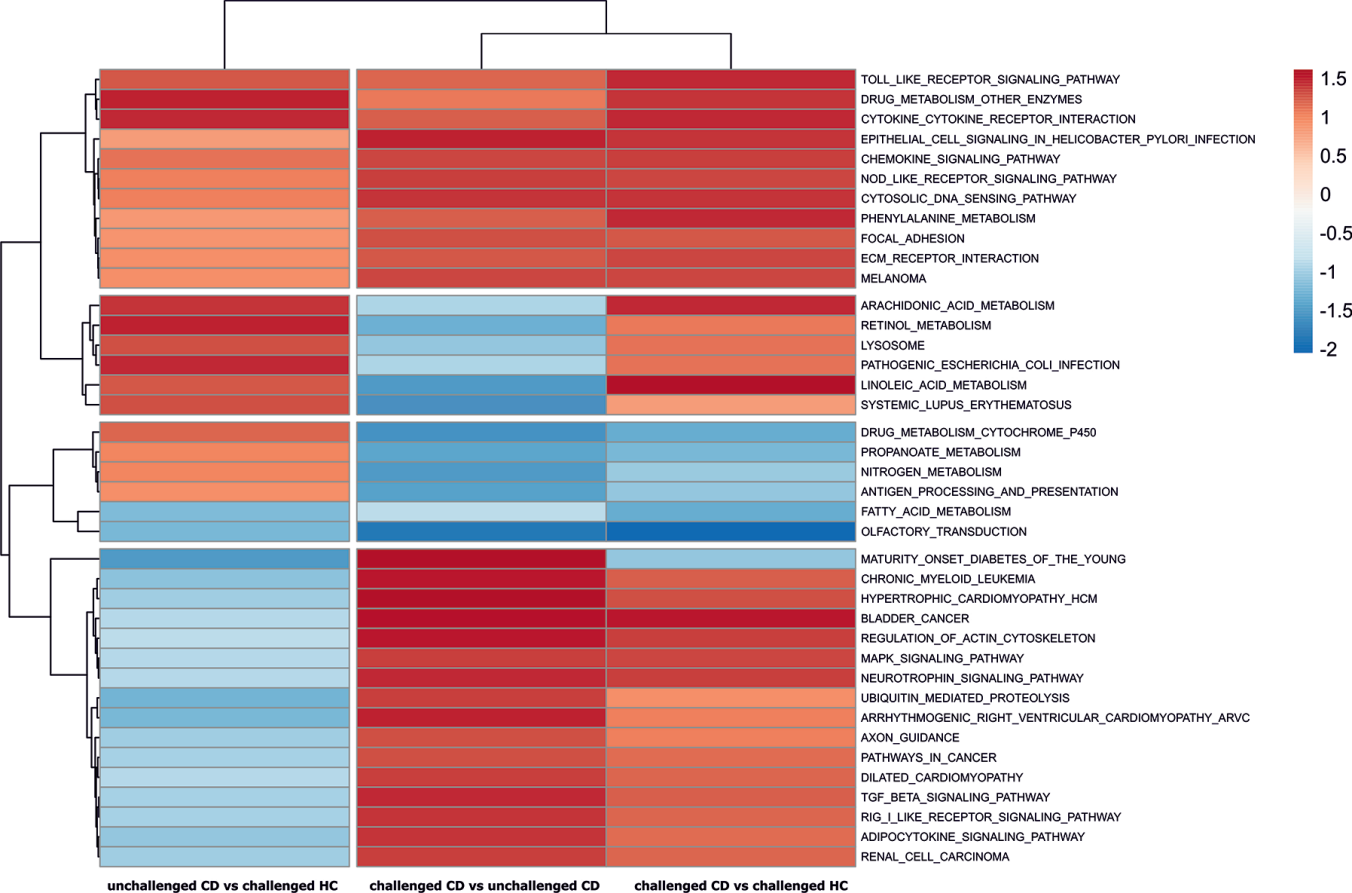
Pathways with significant enrichment scores from GSEA analyses. The normalized pathway enrichment scores (NES) of significantly perturbed KEGG pathways (p-values < 0.05) is used to draw a heat map across the three analyses. Row clustering of the KEGG pathways groups the pathways into CD relevant pathways regardless of treatment, and gluten exposure associated pathways in patients.

We used gene set enrichment analysis with KEGG pathways to gain unbiased insight of CD relevant pathways. Clustering of all the considered KEGG pathways using their obtained normalized enrichment scores in the three analyses gave a global picture of the pathways that show a tendency for upregulated enrichment in CD patients regardless of treatment, and pathways that are specifically affected by the short gluten exposure in patients (**Figure S3**, for a complete list of GSEA analysis result see **Supplementary File S3**). Pathways such as toll-like receptor signaling, cytokine-cytokine receptor interaction, Nod-like receptor signaling, and tight junction showed tendency for upregulation in all three analyses including unchallenged CD vs challenged HC, suggesting that they are perhaps a persistent feature of CD, relevant in both treated CD as well as CD during gluten exposure. Other pathways like natural killer cell mediated cytotoxicity, MAPK and T cell receptor signaling appear to be specifically perturbed towards upregulation only in gluten exposed conditions (challenged CD vs unchallenged CD and challenged CD vs challenged HC) and generally not in treated patients (unchallenged CD vs challenged HC). On the other hand, pathways such as Arachidonic acid, Linoleic acid, Retinol metabolism and pathogenic Escherichia coli infection appear to be down regulated or with no difference in challenged CD vs unchallenged CD, but show tendency for upregulation in patients compared to healthy controls regardless of treatment (**Figure S3**), suggesting that they are already perturbed in patients compared to heathy controls despite their downregulation in gluten exposed compared to treated patients. Statistically significant enrichment scores were detected for 39 KEGG pathways in at least one of the analyses (**Figure 2**). Similarly, the significantly enriched pathways can generally be grouped into 1) those that tend to be upregulated in all three analysis, i.e., in treated and untreated patients compared to controls or treated patients, such as cytokine-cytokine receptor interaction and toll-like receptor signaling, 2) those that are specifically upregulated following gluten exposure and not in GFD patients such as regulation of Actin cytoskeleton, and 3) those that are only upregulated both in treated and untreated patients compared to controls but not between gluten exposed and unexposed patients such as arachidonic acid metabolism. Only cytokine-cytokine receptor interaction with upregulation and olfactory transduction with downregulation were detected with statistical significance in all three analyses.

**Figure 3:**
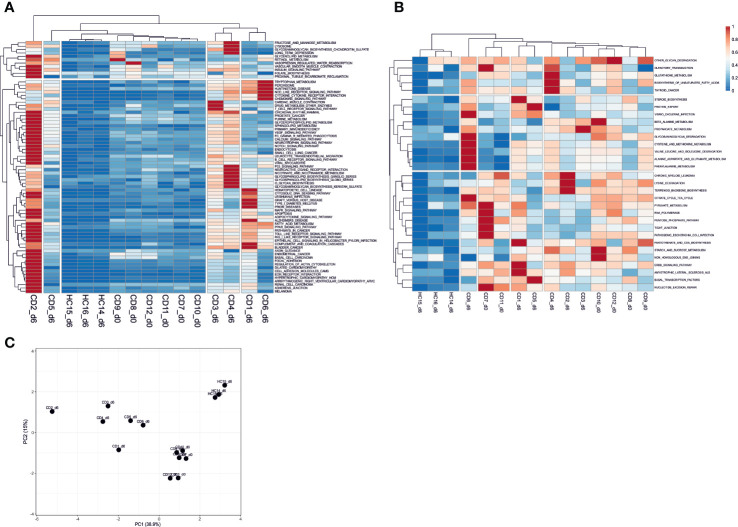
Sample-specific pathway deregulation analysis. **(A)** pathways in Cluster IV that appear “normalized” in treated day 0 (unchallenged) CD patient samples with pathway deregulation scores (PDS) close to the average in the healthy samples is shown. These pathways are deregulated in gluten exposed day 6 CD samples. **(B)** Pathways in Cluster II that show constitutive deregulation in CD patients regardless of treatment are shown. Clustering of the samples based on their PDS across pathways shows the similarity of samples over these pathways. Clusters IV and II are shown here, for the complete result see **Figure S3**. **(C)** PCA plot of the complete sample versus pathways deregulation score matrix data. The PCA shows clear separation of sample types by gluten exposure and health status. Gluten exposed CD patient samples have the most within cluster variation. CD, celiac disease; HC, healthy control; d0, day0; d6, day6.

We explored the KEGG pathways further in sample-specific manner using Pathifier to evaluate their pathway deregulation scores (PDS) from the “healthy” state (see **Supplementary File S4**). Clustering analysis showed samples formed three clusters of PDS profiles containing healthy controls, treated CD, and gluten challenged CD groups. The gluten exposed patient samples show the most variation in the PDS space but are clearly separated from the unchallenged CD and challenged HC samples, while the latter sample groups are closer to each other (**Figure S4** and **Figure 3C**). Unchallenged CD samples are in between the healthy state and gluten exposed patients on the principal component 1 axis, explaining close to 40% of the variation in the data (**Figure 3C**). Clustering of the pathways using their scores across samples provides insight of constitutively deregulated pathways in CD regardless of treatment, and pathways that are likely “normalized” by GFD treatment (see **Supplementary Figure S4**). Cluster IV and cluster II are shown as representatives of treatment “normalized” and constitutively deregulated pathways respectively on **Figure 3A, B** (see **Figure S4**). Further clustering of the samples using only Cluster IV data shows clear separation of gluten challenged CD samples from unchallenged CD and challenged HC (**Figure 3A**), suggesting that these pathways have “normalized” following treatment; while for cluster II on **Figure 3B**, challenged HC samples are clearly separate while challenged and unchallenged CD samples are not clearly disjointed, suggesting that these pathways remain deregulated in treated CD patients. Generally, signaling pathways involved in the autoimmune immune response appear to have “normalized” on GFD while amino acid metabolic pathways such as cysteine and methionine, glutathione, alanine aspartate and glutamate metabolism, as well as other interesting pathways such as tight junction, pathogenic Escherichia coli infection and olfactory transduction appear to be largely constitutively deregulated or perturbed regardless of treatment.

### Upstream Regulator Predicts Constitutively Activated Mediators

We also used the upstream regulator prediction method in IPA to examine possible genomic regulators that can help explain the pattern of differential expression observed in our dataset. The method predicted previously known mediators of the immunological response in CD including genes such as IFNG which were detected with low reads in our dataset (and were consequently not called differentially expressed, see **Table S2**) despite their well-established upregulation in CD and increased overall expression in blood of CD patients 6 days following gluten challenge (14). Among gluten-exposed patients, cytokines such as TNF, IFNG, and IL1B, as well as Toll-like receptors such as TLR4, TLR3, and TLR9 implicating the innate immune response were predicted to be activated. Selected upstream regulators predicted to be activated are shown on **Figure S5A**. A detailed list of all predicted upstream regulators is provided in the **Supplementary File S2**.

Particularly interesting were predicted upstream regulators with significant p-values from unchallenged CD vs challenged HC DEGs that were also predicted to be significant both from the challenged CD vs unchallenged CD and challenged CD vs challenged HC DEGs, and showed z-scores very close to the activation cut-off (z-score >2 for activation or <-2 for inhibition, see Methods, **Figure S5B**). These included genes NFE2L2 (Nuclear Factor, Erythroid 2 like 2), SMARCA4 (SWI/SNF Related, Matrix Associated, Actin Dependent Regulator Of Chromatin), TP53 (tumor protein p53), JUN (Jun Proto-Oncogene, AP-1 Transcription Factor Subunit), CSF2 (colony stimulating factor 2), and FOS (Fos proto-oncogene, AP-1 transcription factor subunit). Some of these predicted upstream regulators appeared to have constitutive activation in CD patients irrespective of gluten exposure status (**Figure S5B**), and are interestingly in locations relatively proximal to previously reported CD associated genetic loci (29). NFE2L2 is on 2q31.2, ~3Mb away from CD associated locus 2q31.3, a locus harboring CD associated SNP on a long intergenic non-coding RNA (lincRNA) that has been shown to have increased expression in active CD biopsies (31). SMARCA4 is on 19p13.2, millions of bases away from CD risk loci 19p13.11 (32, 33). The gene JUN is on 1p32.1, ~2Mb off CD associated loci 1p31.3, while FOS is on 14q24.3 with another CD association on 14q24.1. Despite its lower z-score in unchallenged CD versus challenged HC comparison, CSF2 is also an interesting predicted regulator as it is on 5q31 region for which CD linkage has be reported but no SNP association has been found (34). Except NFE2L2, which was significantly upregulated in the challenged CD vs unchallenged CD analysis, none of the interesting predicted regulators genes on **Figure S5B** were directly observed to have differential expression in our dataset. The prediction of these interesting constitutively activated upstream regulators was not due to simple sharing of downstream DE genes in our dataset, as IPA has used largely unique set of DE genes to predict each as possible regulator (**Figure S6A**), and the possibility of biologically relevant protein-protein interaction among them is significant as predicted by String (**Figure S6B**) (35).

### Genes With Constitutively Altered Expression

Some genes showed constitutively altered expression in both challenged and unchallenged patients compared to the challenged healthy controls, while having no difference in expression between challenged versus unchallenged patient samples (**Table 2**). These genes may signify an important expressional or molecular state among patients that remains constant regardless of gluten intake. All of these genes showed upregulation in patients compared to healthy controls, and both their individual and collective expressions clearly distinguish patients from the gluten exposed healthy controls (**Figure S7**). Interestingly, some of these genes are on previously reported CD associated loci. CSTB is on locus 21q22.3, a region with previous CD implicated genes UBASH3A and ICOSLG (36, 38); MEF2BNB-MEF2B (BORCS8-MEF2B) is on 19p13.11, a locus harboring MYO9B with linkage to CD but with population-specific SNP variant association signals (33, 37); ARL11 and AARSD1 are on CD loci 13q14.2 and 17q21.31 respectively with no candidate genes previously identified (36).

**Table 2:** Genes with constitutively altered expression in CD patient PBMCs.

Gene	Full gene name	Biological function	Location	On CD locus	Candidate genes	Refs
FAU	Ubiquitin-Like Protein Fubi And Ribosomal Protein S30	Component of cytoplasmic ribosome; antimicrobial activity	11q13.1			
ACTG1	actin gamma 1	structural constituent of cytoskeleton; cell motility	17q25.3			
GSTP1	glutathione S-transferase pi 1	xenobiotic metabolism, detoxification	11q13.2			
ALDOA	aldolase A, fructose-bisphosphate	Plays a key role in glycolysis and gluconeogenesis	16p11.2			
CSTB	cystatin B (stefin B)	cysteine protease inhibitors, protecting against proteases leaking from lysosomes	21q22.3	21q22.3 (rs4819388)	UBASH3A, ICOSLG	(36)
POLR2L	polymerase (RNA) II (DNA directed) polypeptide L	encodes a subunit of RNA polymerase II	11p15.5			
CRIP1	cysteine-rich protein 1 (intestinal)	zinc transport	14q32.33			
TUBB4B	tubulin, beta 4B class IVb	Structural constituent of cytoskeleton;mitosis,protein transport	9q34.3			
ARPC4-TTLL3	ARPC4-TTLL3 (actin related protein 2/3 complex, subunit 4 & tubulin tyrosine ligase-like family, member 3) readthrough	Actin filament polymerization	3p25.3			
CCT7	chaperonin containing TCP1, subunit 7 (eta)	assists the folding of proteins	2p13.2			
PQBP1	polyglutamine binding protein 1	transcription activation	Xp11			
MRPL54	mitochondrial ribosomal protein L54	protein synthesis	19p13.3			
RAB13	RAB13, member RAS oncogene family	polarized transport, assembly and/or the activity of tight junctions	1q21.3			
ARL11	ADP-ribosylation factor-like 11	Tumor suppressor, Apoptosis	13q14.2	13q14.2 (rs2762051)		(36)
MEF2BNB-MEF2B (BORCS8-MEF2B)	MEF2BNB-MEF2B (Myocyte Enhancer Factor 2B) readthrough	Transcription regulation; likely involved nonsense-mediated decay (getting rid of early truncated proteins); expressed highly in lymph nodes	19p13.11	19p13.11 (rs2305764)	MYO9B	(33, 37)
AARSD1	alanyl-tRNA synthetase domain containing 1	tRNA metabolic process	17q21.31	17q21.31 (rs2074404)		(36)

## Discussion

We explored the whole PBMC transcriptome of treated and short 3-day gluten challenged CD patients in comparison to non-CD gluten exposed healthy controls using RNA sequencing. Short *in vivo* gluten exposure generally induced altered transcriptomic signature in CD patient PBMCs compared to both treated patients and healthy controls. Distinct transcriptional profile was also observed in treated CD patients compared to gluten consuming healthy controls, indicating that persistent transcriptional alteration is a feature of CD despite GFD treatment and remission of clinical symptoms. We also observed some interesting exceptions, with around a third of gluten-challenged patient samples appearing closer to gluten challenged healthy controls, and a third of gluten unchallenged patient samples clustering with gluten challenged patient samples. Such exceptions and variation has previously been reported (10). Possible reasons for such exceptions could range from differences in GFD adherence to other possible pre-experiment infections, as well as due to the high clinical and molecular variation among CD patients, for instance in the severe cytokine release syndrome after gluten exposure and the possible attenuation of the cytokine release intensity with each successive gluten exposure (39, 40).

Among the 79 CD associated candidate genes (29), we detected significant transcriptional changes in GLB1 (3p22.3, downregulated), CCR2 (3p21.31, downregulated) and TNFAIP3 (6q23.3, upregulated) following gluten exposure in patients. This is much lower than what has been reported by Quinn et al. (detected 25 CD associated genes DE in CD patient blood *in-vitro* stimulated CD4+ T-cells) and Bragde et al. (detected 21 CD associated genes DE in CD patient intestinal biopsy samples) (10, 11), yet noteworthy as it provides insights of *in-vivo* molecular changes in patient PBMC using the gluten challenge study model. While CCR2 was reported previously to have altered expression in CD patient blood and intestinal biopsy samples in the recent studies (10, 11), gluten-induced differential expression of GLB1 (Galactosidase Beta 1) and TNFAIP3 (Tumor Necrosis Factor Alpha-Induced Protein 3) in our dataset is a new finding and provides further evidence of their relevance in CD. The differential expression of these CD candidate genes in our PBMC dataset and not in the studies by Quinn et al. and Bragde et al., both with higher sample sizes and deeper sequencing than the current study, underscored the effect of tissue, cell type, and study design, leading to different reports, in some cases discrepant, with varying versions of the complete detail of the transcriptome in CD. For such reasons, pathway analysis provides a more comprehensive picture of the transcriptome in CD.

The inhibition of Peroxisome proliferator-activated receptor (PPAR) signaling in CD mucosa has been previously reported (41, 42), and our IPA result shows gluten induces its inhibition among cells in the PBMC. We also found inhibition of LXR/RXR activation which is involved in lipid metabolism and inflammation and has been shown to be important in increasing the phagocytic capacity of macrophages (43). Its inhibition upon gluten exposure might indicate reduced capacity to control inflammation. Of note, DEGs in treated patients compared to healthy controls were enriched with IPA pathways suggesting abnormal activity of cytoskeletal and cell-to-cell adheren function despite a relatively long period of GFD treatment averaging more than 5 years.

As expected and was reported from recent studies that assessed KEGG pathways in CD (10, 12), CD patients showed upregulation of cytokine-cytokine receptor interaction and toll-like receptor signaling when exposed to even short 3-day gluten exposure. While decreased retinol metabolism was also detected as was also reported by Leonard et al. (12) in gluten exposed compared to unexposed patients, retinol metabolism generally tended towards constitutive upregulation in CD patients compared to controls. This was also the case for arachidonic acid metabolism, lysosome, linoleic acid metabolism, and E.coli infection pathways. This result supports the view of unsaturated fatty acid metabolic impairment in CD as well as the constitutive activation of infection-induced pathways (specifically, altered innate immune response pathways due to stress from recurring infections), perhaps due to the reported increased prevalence of enterotoxigenic escherichia coli among CD patients early on in life (44, 45). The significant downregulation of Olfactory pathway was also another interesting finding. Olfactory dysfunction has been reported to have association with autoimmune diseases (46), with for instance downregulation of olfactory signal transduction genes observed in animal models of Multiple Sclerosis (47). Furthermore, our assessment of pathways in each individual sample combined with PCA analysis showed GFD treated patients had deregulated pathways and showed distinct combined overall biological pathway profile from the “healthy” pathway status observed in gluten exposed HLA-DQ2+ healthy controls. This result is consistent with recent reports of Sangineto et al. (48) who have showed constitutive changes in CD PBMC despite long-term GFD treatment, albeit compared to non-DQ2+ healthy controls, and A.James et al. (49) who have showed that majority of long-term GFD treated CD patients have persistent villous atrophy despite being seronegative for transglutaminase antibodies. Glutathione is an antioxidant and its proper production and levels are necessary in CD as oxidative stress is a major feature in CD intestinal lesions. Our data shows deregulation and increased constitutive upregulation of Glutathione metabolism while previous studies have shown decreased concentrations of Glutathione in CD mucosa and peripheral blood (50–52). This might indicate overall failure in CD patients to produce enough antioxidants despite increased metabolism, perhaps as a result of the overall systemically destabilized amino acid levels in celiac disease (53).

CD associated proinflammatory cytokines such as IFNG and IL2 were not detected with enough mRNAs in all samples in our experiment and consequently were not DE. Apart from our shallow sequencing, this could be due to the low expression levels of cytokine genes and overall composition of PBMC cells, transient expression of such genes, and/or low sensitivity of the STRT RNAseq method used. Nonetheless, upstream regulator prediction showed the increase in their signatures already with short *in-vivo* gluten challenge, confirming reports of systemic increase in the expression of pro-inflammatory cytokines (39). Interestingly, the upstream regulators that appear to be constitutively activated in CD, namely, NFE2L2, SMARCA4, JUN, FOS, and CSF2 were within millions of bases to previously reported CD associated loci. Although somewhat distant for cis-regulation, the repeated occurrence of these predicted regulators near to CD associated loci is unlikely to be by chance. Possible explanations could be distal regulation due to chromatin conformation and interactions (31), or involvement in the same biological mechanism of both the predicted regulator and the genetic element in the CD loci thus exerting similar effects on the downstream genes that we detected as DE, or simply due to the involvement of another genetic factor affecting the region of the chromosome harboring both and contributing to CD development. SMARCA4 (BRG1) on 19p13.2 and CSF2 on 5q31 region are particularly interesting as they are near regions showing strong linkage to CD for which only population-specific and/or multiple associations with moderate signals have been reported (33, 37, 54). SMARCA4 is part of the chromatin remodeling complex SWI/SNF with transcriptional regulation effects and is implicated with diseases such as myocardial infarction and the self-renewal capacity of cancer cells, in which it plays a direct role in sustaining oncogenic transcriptional activity (55, 56). Given that myocardial infarction and small intestinal cancer occur more frequently among CD patients (57, 58), SMARCA4 is a good CD candidate gene for the 19p13 locus. Increased expression of the colony-stimulating factor, CSF2, in the healing CD mucosa has also been previously reported (59), further indicating the constitutive involvement of the 5q31 locus in CD pathology. Another interesting upstream regulator with constitutive effect in CD is the gene JUN, whose product is a component of the activator protein-1 (AP-1), a transcription factor of various target genes regulating cellular functions such as proliferation and apoptosis. JUN has been shown to be upregulated in CD mucosa and plays a central role in the impairment of the T regulatory effects of TGF-β in CD secondary to the IL-15 (60, 61). Constitutive activation of Epidermal Growth Factor Receptor (EGFR) and Extracellular Signal Regulated Kinase (ERK), both upstream of the signaling cascade that activates AP-1, has also been reported in CD intestinal biopsies and even skin fibroblasts maintaining increased crypt proliferation in CD mucosa (62). AP-1 and its constituent JUN have also been implicated in tight junctional function (63, 64) as well as virus-induced cellular function alteration (65), all pointing to the central constitutive role of JUN in CD pathology.

We also detected genes from previously reported CD associated loci that showed significant upregulation in patients only when compared to healthy controls. Among these, MEF2BNB-MEF2B (BORCS8-MEF2B) on 19p13.11 has an interesting possible function. It is a read-through transcript which generally are believed to have involvement in nonsense-mediated decay (NMD) (66). Its constituent MEF2B was reported to be a target of Epstein-Barr Virus proteins and plays a role in the survival of virus-infected B-cells maintaining latent infection (67). Epstein-Barr virus protein occupied genomic loci have also been implicated to intersect with CD associated loci (68), while virus proteins can use read-throughs to protect their RNAs from control by NMD (69). Although the BORCS8-MEF2B transcript was detected with very low numbers in CD patients (**Figure S7b**), it was not detected in all healthy controls and may thus be only expressed when mRNA decay is required or as a result of possible latent infection.

Our study has some limitations mainly due to the small sample size and unavailability of samples for some of the patients and healthy controls. The samples were selected based on availability of enough stored PBMC and good quality RNA from a larger cohort collected earlier for other studies (day 0 samples for the healthy controls and patients CD1, 2 & 5, and day 6 samples for patients CD9, 10 & 12 were missing due to sample unavailability). As a result, we were unable to conclusively explain the clustering pattern of the samples with “exceptional” transcriptomic profiles on **Figure S1**, i.e, day 0 samples of CD3, 4 & 6, and day 6 samples of CD7, 8 & 11. As described in the result section, a closer look at the clinical features of these samples showed patients CD3, 4 & 6 had higher proportion of heightened immune response status, in some cases unrelated to CD, already at day 0, while day 6 samples of CD7, 8 & 11 had higher proportions of no symptom or IFNg release upon gluten challenge. Particularly, the case of day 6 samples of CD7, 8 & 11 was interestingly similar to the report by Quinn et al. (10) in which a third of CD patients showed CD4+ T-cell transcriptomic profiles similar to HLA-unmatched healthy controls (see **Figure 1** of Quinn et al.). Additionally, evaluation of sample-specific pathway de-regulation in the “exceptional” samples seems to suggest 1) day 0 samples of CD3, 4 & 6 have increased deregulation in immune response related pathways similar to day 6 samples, confirming our heightened immune response hypothesis (cluster II in **Figure S8**) 2) day 6 samples of CD7, 8 & 11 are not necessarily similar to the day 6 healthy controls, with particularly day 6 samples of CD7 and CD11 showing deregulation profiles closer to other patient day 0 samples (**Figure S8**), perhaps requiring longer than 3 days to study gluten’s effect in such patients. Given the high heterogeneity of CD, with such patterns possibly arising from confounding or uncontrolled factors and their likely pervasive impact with our small sample size, we chose to exclude these “exceptional” samples from downstream analyses. We recognize that there could be alternative interpretations of the clustering profile seen on **Figure S1** in which all day 0 samples can be taken as representing two CD subtypes on GFD, with the first subtype being the cluster containing day 0 samples of CD3, 4 and 6 and the shift to the right, to their day 6 samples, representing the current gluten challenge effect, and day 0 samples of CD7–12 representing another subtype in which gluten-challenge induces the same effects as in healthy controls. In such a scenario, our report of constitutive alterations holds true only in cases where the reported changes happened in both the treated day 0 and the gluten challenged day 6 samples compared to the healthy controls. In future studies with larger sample sizes, if such “exceptional” samples still show the same pattern despite controlling for all other factors, this could be utilized to design gene-expression profile based diagnosis and monitoring of CD and treatment status.

In conclusion, a short 3-day oral gluten challenge induced systemic transcriptional changes. While GFD treatment normalizes immune response pathways and most consequently clinical symptoms, treated CD patients still maintain an altered biological pathway profile even compared to gluten exposed healthy controls that is mainly characterized by deregulated amino acid metabolism and tight junction as well as or due to possible microbial infection. We find some genes involved constitutively in CD pathology regardless of treatment from CD associated loci, and in some cases should be considered good candidates for loci for which evidence for SNP association despite strong linkage, or for which candidate genes despite SNP association have been scant.

## Data Availability Statement

The sequence datasets generated in this study have been deposited at the European Genome-phenome Archive (EGA) and can be accessed under the accession number EGAS00001004860.

## Ethics Statement

The studies involving human participants were reviewed and approved by The Ethical Committee of the Pirkanmaa Hospital District, Finland. The patients/participants provided their written informed consent to participate in this study.

## Author Contributions

Study concept and design: PS. Contribution to study design: DY, AK, RA. Acquisition of study samples, technical and material support: KKa, KKu, AK, RA, MM, SL. WetLab sample processing: PS. Data analysis and interpretation: DY, PS. Statistical analysis: DY, DG, PS. Manuscript drafting: DY, PS. Critical revision of manuscript: KKa, KKu, MM, RPA, DG. Study supervision: PS, DG. All authors contributed to the article and approved the submitted version.

## Funding

This work was supported by the Academy of Finland, European Commission (Marie Curie Excellence Grant), Sigrid Juselius Foundation, the Competitive State Research Financing of the Expert Area of Tampere University Hospital, and by SalWe Research Programs INTELLIGENT MONITORING and GET IT DONE (Tekes - the Finnish Funding Agency for Technology and Innovation grants 648/10 and 3986/31/2013).

## Conflict of Interest

The authors declare that the research was conducted in the absence of any commercial or financial relationships that could be construed as a potential conflict of interest.
