# Supplementary material for: Effects of In Vivo Gluten Challenge on PBMC Gene Expression Profiles in Diet Treated Celiac Disease
Source: Front Immunol. 2020 Dec 11;11:594243. doi: 10.3389/fimmu.2020.594243 (PMC7759501; doi:10.3389/fimmu.2020.594243)
Supplement: Supplementary file 1 [file Presentation_1.pdf]

Effects of *in vivo* gluten challenge on PBMC gene expression profiles in diet treated celiac disease

Dawit A Yohannes[1,2], Andrea de Kauwe[1,2], Katri Kaukinen[3], Kalle Kurppa[4,5], Markku Mäki[4], Robert P Anderson[6], Sten Linnarsson[7], Dario Greco[8,9] and Päivi Saavalainen[1,2,*]

[1] Research Programs Unit, Translational Immunology, University of Helsinki, Helsinki, Finland

[2] Department of Medical and Clinical Genetics, University of Helsinki, Helsinki, Finland

[3] Department of Internal Medicine, Tampere University Hospital and Faculty of Medicine and Health Technology, Tampere University, Tampere, Finland

[4] Department of Pediatrics, Tampere University Hospital, Tampere, Finland, Center for Child Health Research, Tampere University, Tampere, Finland,

[5] Seinäjoki University Consortium and Seinäjoki Central Hospital, Seinäjoki, Finland

[6] Walter and Eliza Hall Institute of Medical Research, Melbourne, Australia

[7] Laboratory for Molecular Neurobiology, Department of Medical Biochemistry and Biophysics, Karolinska Institute, Stockholm, Sweden

[8] Faculty of Medicine and Health Technology & BioMediTech Institute, Tampere University, Tampere, Finland

[9] Institute of Biotechnology, University of Helsinki, Helsinki, Finland

* Corresponding Author: paivi.saavalainen@helsinki.fi

Table S1: Details characteristics of study subjects. For each patient and healthy control, sex, age, GFD duration is given. Yellow highlighted samples were excluded from downstream analysis.

SampleID	Condition	TimePoint	Sex	Age	hlaDQ	GFDduration(yrs or wks)	IFNg ELISpot	Symptoms upon challenge
CD7_d0	CD	day0	F	41	DQ2	3	none	flatulence and loose stools during challenge
CD8_d0	CD	day0	F	66	DQ2	2	weak	no symptoms
CD9_d0	CD	day0	F	48	DQ8	NA	NA	Did not undergo challenge
CD10_d0	CD	day0	F	22	DQ2	1	weak	urticaria (hives) during challenge
CD11_d0	CD	day0	M	55	DQ2	10	strong	no symptoms
CD12_d0	CD	day0	M	63	DQ2	14	None	loose stools during the challenge
CD4_d0	CD	day0	M	50	DQ2	3	weak	loose stools & nausea on day 3 (had flu/fever during week)
CD6_d0	CD	day0	F	61	DQ2	16	Strong	no symptoms, but gastroenteritis the week previous
CD3_d0	CD	day0	F	37	DQ2	10	weak	nausea & vomitting, unable to complete full challenge
CD1_d6	CD	day6	M	68	DQ2	16	weak	mild abdominal pain & loose stools on day 3; resolved post-challenge
CD2_d6	CD	day6	F	63	DQ2	8	weak	mild dyspepsia (heartburn) on day 1, then totally asymptomatic
CD3_d6	CD	day6	F	37	DQ2	10	weak	nausea & vomitting, unable to complete full challenge
CD4_d6	CD	day6	M	50	DQ2	3	weak	loose stools & nausea on day 3 (had flu/fever during week)
CD5_d6	CD	day6	F	61	DQ2	1	Strong	no symptoms
CD6_d6	CD	day6	F	61	DQ2	16	Strong	no symptoms, but gastroenteritis the week previous
CD7_d6	CD	day6	F	41	DQ2	3	none	flatulence and loose stools during challenge
CD8_d6	CD	day6	F	66	DQ2	2	weak	no symptoms
CD11_d6	CD	day6	M	55	DQ2	10	strong	no symptoms
HC15_d6	HC	day6	F	45	DQ2	4wks	none	abdominal distension
HC16_d6	HC	day6	F	41	DQ2	4wks	none	abdominal distension
HC14_d6	HC	day6	F	30	DQ2	4wks	none	abdominal distension

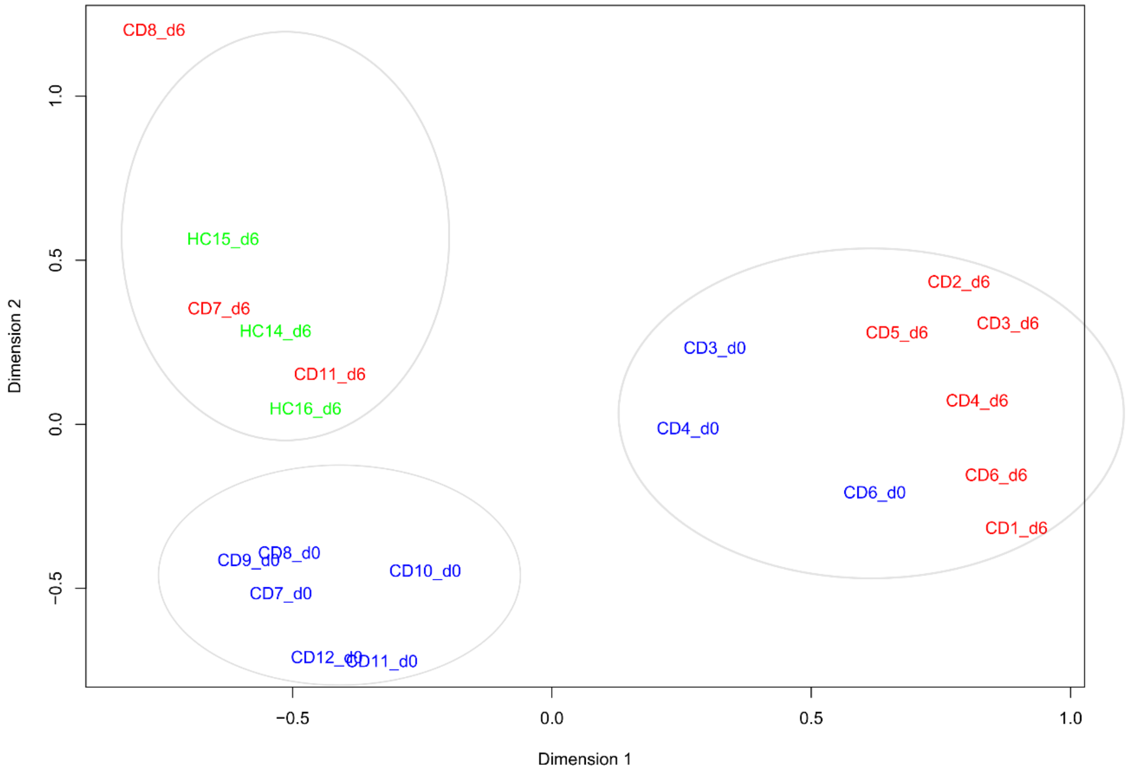

Figure S1: Multidimensional scaling (MDS) plot of the total gene expression profile of all samples: gluten unchallenged patient samples from day 0 are shown in blue. Gluten challenged patient and healthy control day six samples are shown in red and green respectively. Day 0 samples of patients CD3,CD4 and CD6 appear to cluster with day 6 samples, while day 6 samples of patients CD7,8 and 11 appear to cluster with healthy controls (or the day 0 side in dimension 1). These samples were excluded from further analysis (see Table S1).

a)

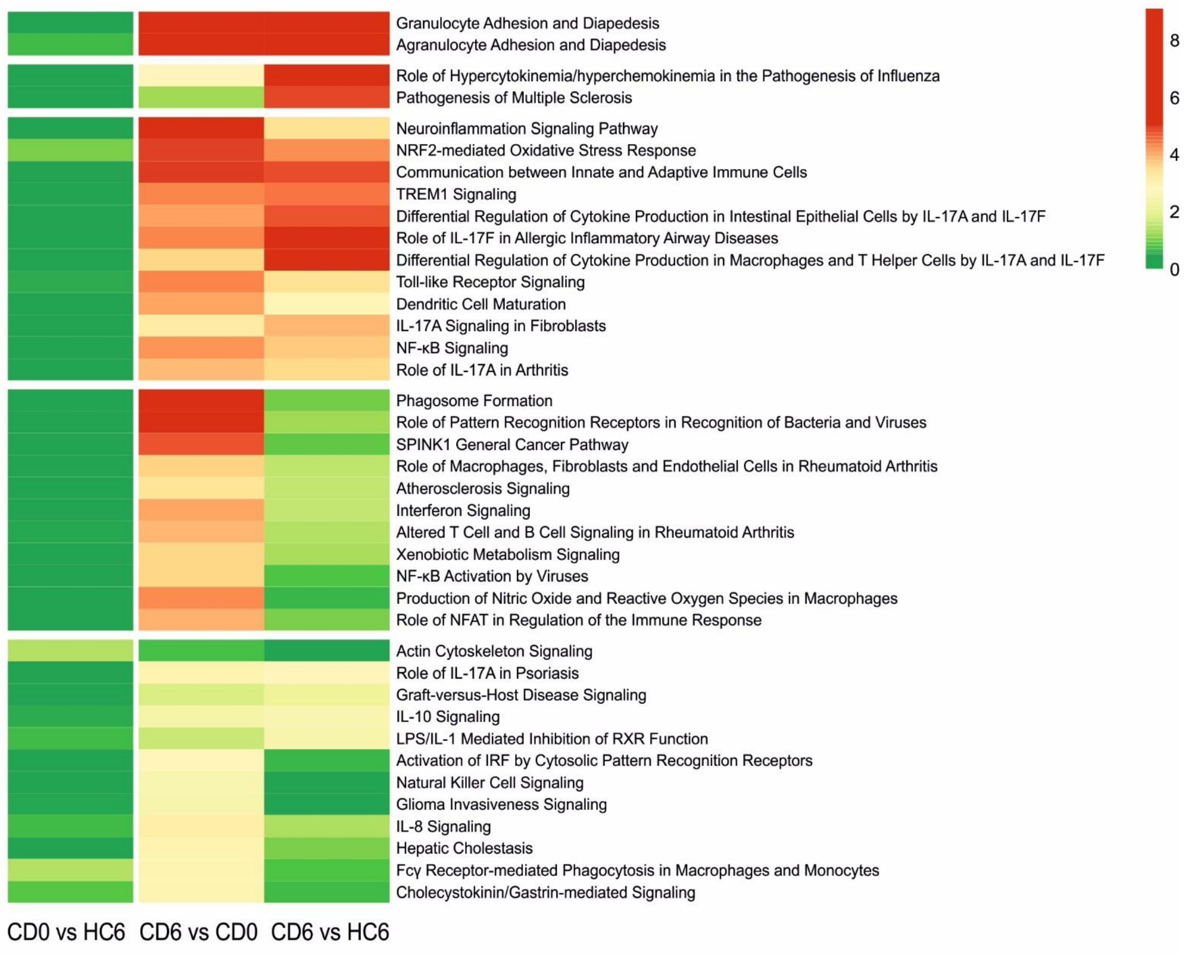

b)

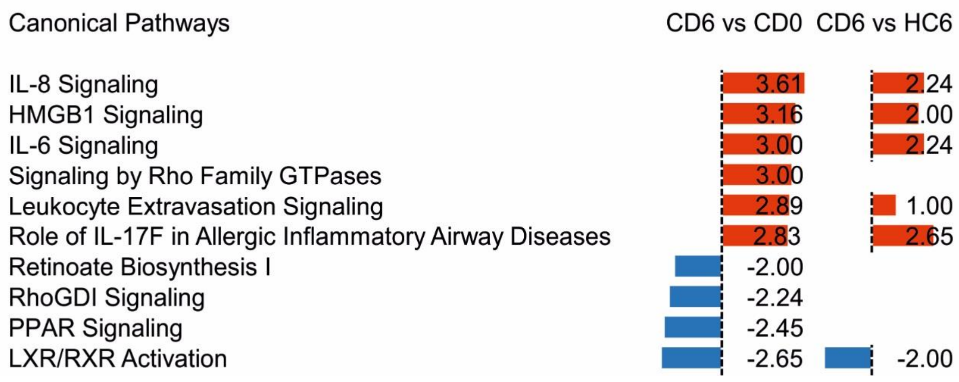

Figure S2: CD associated IPA canonical pathways assessed from DEGs of each analyses. a) Selected IPA canonical pathways that were significantly overrepresented among the DEGs of at least one of the three analyses are shown. Colors represent -log10 values of adjusted p-values with cut off for significance being 1.3 (corresponding to p-value < 0.05), green to red color intensity showing low to high pathway enrichment significance. Pathways on the rows are clustered according to their p-value scores across the three analyses. b) Selected pathways predicted to be significantly activated or inhibited are shown along with their z-scores from all three analyses. (Z-score > 2 indicates activation, and < -2 indicates inhibition). CD0, celiac disease samples from day 0 (the unchallenged CD group); CD6, celiac disease samples from day 6 (challenged CD); HC6, healthy control from day 6 (challenged CD).

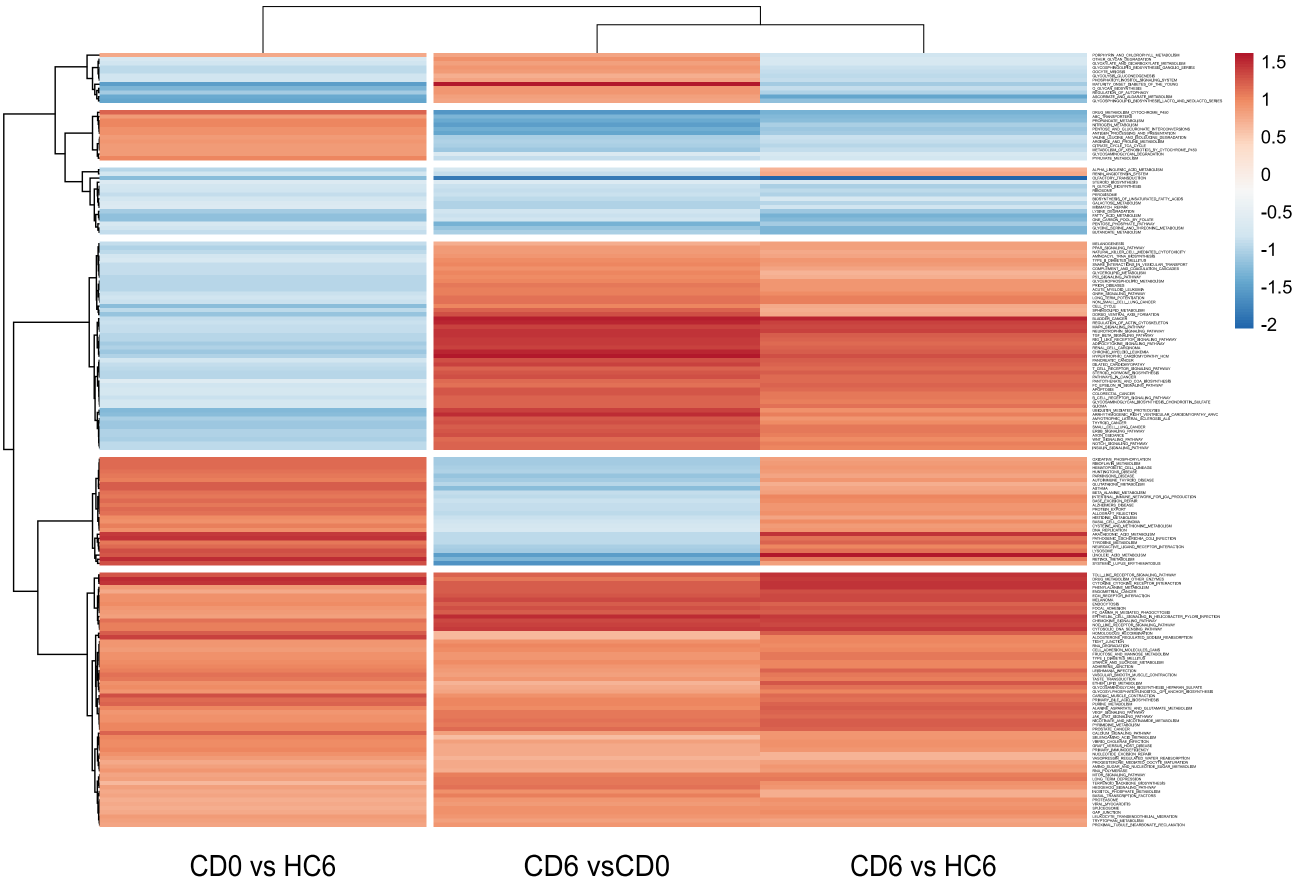

Figure S3: GSEA result heat map. The normalized pathway enrichment scores (NES) of each KEGG is used to draw a heat map of all KEGG pathways evaluated in the three analyses. Row clustering of the KEGG pathways using their NES in the three analyses shows pathways enriched and upregulated in CD regardless of treatment, and pathways specifically upregulated only during the short gluten exposure in patients, both in CD6 vs CD0 (challenged CD vs unchallenged CD) and CD6 vs HC6 (challenged CD vs challenged HC). CD0, celiac disease samples from day 0 (unchallenged CD); CD6, celiac disease samples from day 6 (challenged CD); HC6, healthy control from day 6 (challenged HC).

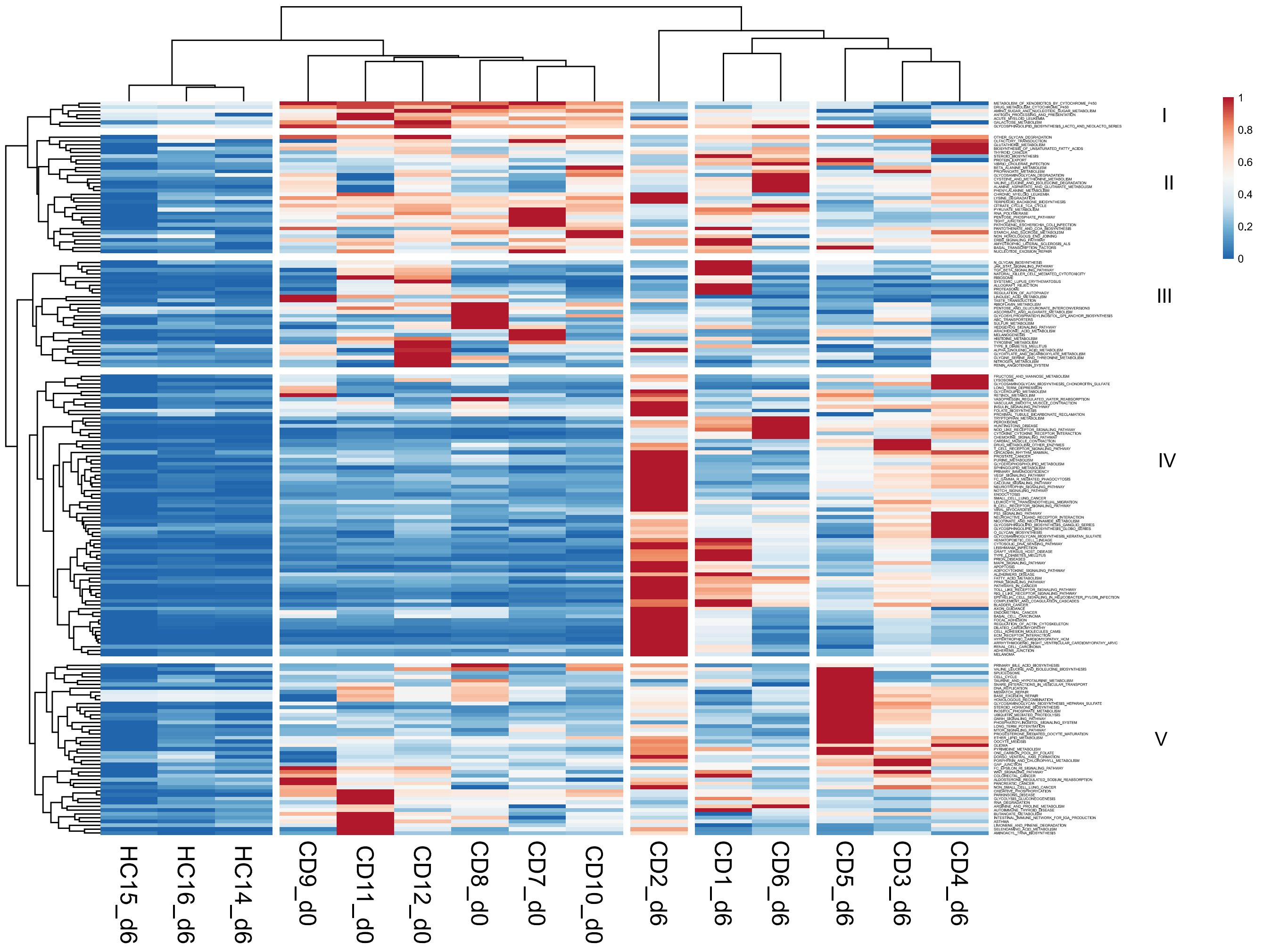

Figure S4: Sample-specific pathway deregulation score heatmap. The distance of each pathway from the "healthy" state is shown using heatmap of all samples. Clustering of the samples based on their PDS over all pathways shows three main branches consistent with their gluten exposure and CD status although treated CD samples are closer to healthy controls. Clustering of pathways helps suggest pathways that are constitutively deregulated in CD patients (clusters II) and treated to normal states (cluster IV). CD, celiac disease; HC, healthy control; d0, day0; d6, day6.

a)

Upstream Regulators	CD6 vs CD0	CD6 vs HC6	CD0 vs HC6
TNF	6.61	5.19	-0.11
NFkB (complex)	5.73	5.56	0.00
IL1B	5.60	5.00	-0.42
IFNA2	5.34	3.86	0.00
IRF7	5.08	3.65	0.00
IFNG	5.08	6.10	0.00
RELA	4.89	3.76	0.00
IFNL1	4.61	3.54	0.00
TLR4	4.43	4.48	0.00
IL1A	4.43	4.13	0.00
TICAM1	4.17	4.09	0.00
IRF5	4.15	2.93	0.00
APP	4.09	3.60	0.00
TLR7	4.07	4.03	0.00
TLR3	3.99	3.64	0.00
PAF1	3.99	2.83	0.00
IL1	3.97	3.72	0.00
F2	3.94	3.52	0.00
TLR9	3.91	4.17	0.00
Interferon alpha	3.74	3.62	0.00
CCL5	3.72	3.33	0.00

b)

Upstream Regulators	CD6 vs CD0	CD6 vs HC6	CD0 vs HC6
NFE2L2	1.00	2.47	1.98
SMARCA4	1.87	3.03	1.96
TP53	0.94	0.62	1.96
JUN	1.68	2.47	1.93
CSF2	2.76	3.28	0.96
FOS	0.84	0.86	0.91

Figure S5: Selected upstream regulators predicted by IPA from DEGs of each analyses. IPA quantifies likely activation or inhibition of regulators using z-score by matching the expression direction of observed DE genes in our dataset known to be regulated by the regulator to the known expression effect in their curated knowledge base. Z-score > 2 indicates activation. Intensity of color shows activation status. Predicted upstream regulators are ordered from highest to lowest z-score based on a) the CD6 vs CD0 (challenged CD vs unchallenged CD) scores and selected regulators are shown, b) the CD0 vs HC6 (unchallenged CD vs challenged HC) scores and selected regulators are shown. CD0, celiac disease samples from day 0 (unchallenged CD); CD6, celiac disease samples from day 6 (challenged CD); HC6, healthy control from day 6 (challenged HC).

a)

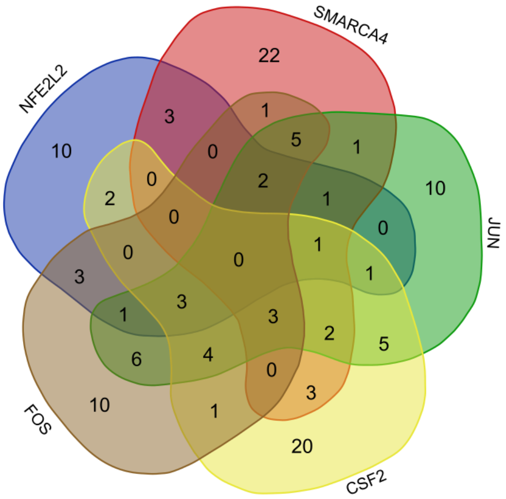

b)

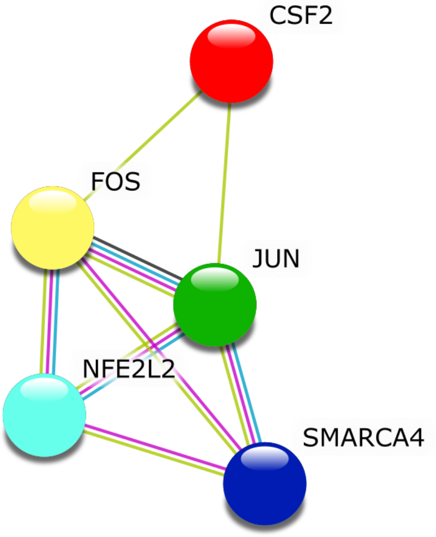

Figure S6: Validity of constitutively activated predicted upstream regulators. a) IPA upstream regulator prediction for the interesting regulators appears to depend largely on unique set downstream DE genes for each regulator, with shared DE genes constituting a smaller portion of the downstream genes used for upstream regulator prediction. b) The constitutively activated upstream regulators have significant enrichment for protein-protein interaction than would be expected by chance if as many proteins from the genome were picked randomly (string PPI enrichment p-value=0.000268;).

a)

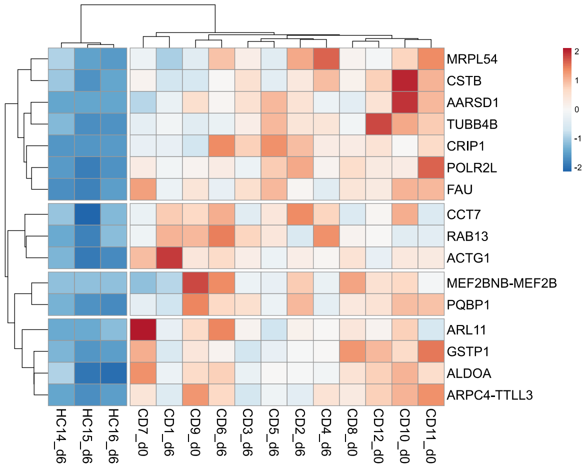

b)

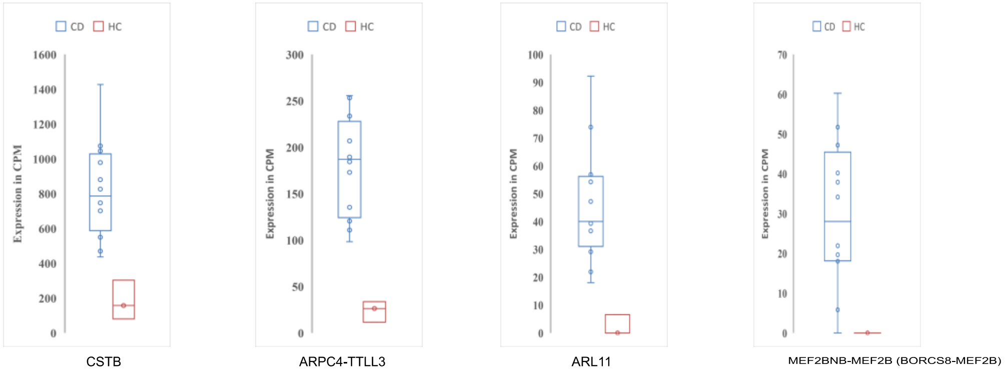

Figure S7: Genes with constitutively altered expression in CD. All genes with statistically significant differential expression in challenged CD vs challenged HC6 and unchallenged CD vs challenged HC but not in challenged CD vs unchallenged CD are shown. a) All of these genes showed upregulation and were able to separate CD patients from healthy controls regardless of treatment. Rows are centered and unit variance scaling is applied to rows in the heatmap. Both genes and samples are clustered using hierarchical clustering with euclidean distance and ward linkage. b) Gene expression in counts per million (cpm) of some of these genes that are in previously reported CD associated loci is shown in the CD patients versus the healthy control samples (from all challenged CD, unchallenged CD and challenged HC samples). ARL11 and BORCS8-MEF2B are relatively lowly expressed in all samples. CD, Celiac disease; HC, healthy control; d0, day0; d6, day6.

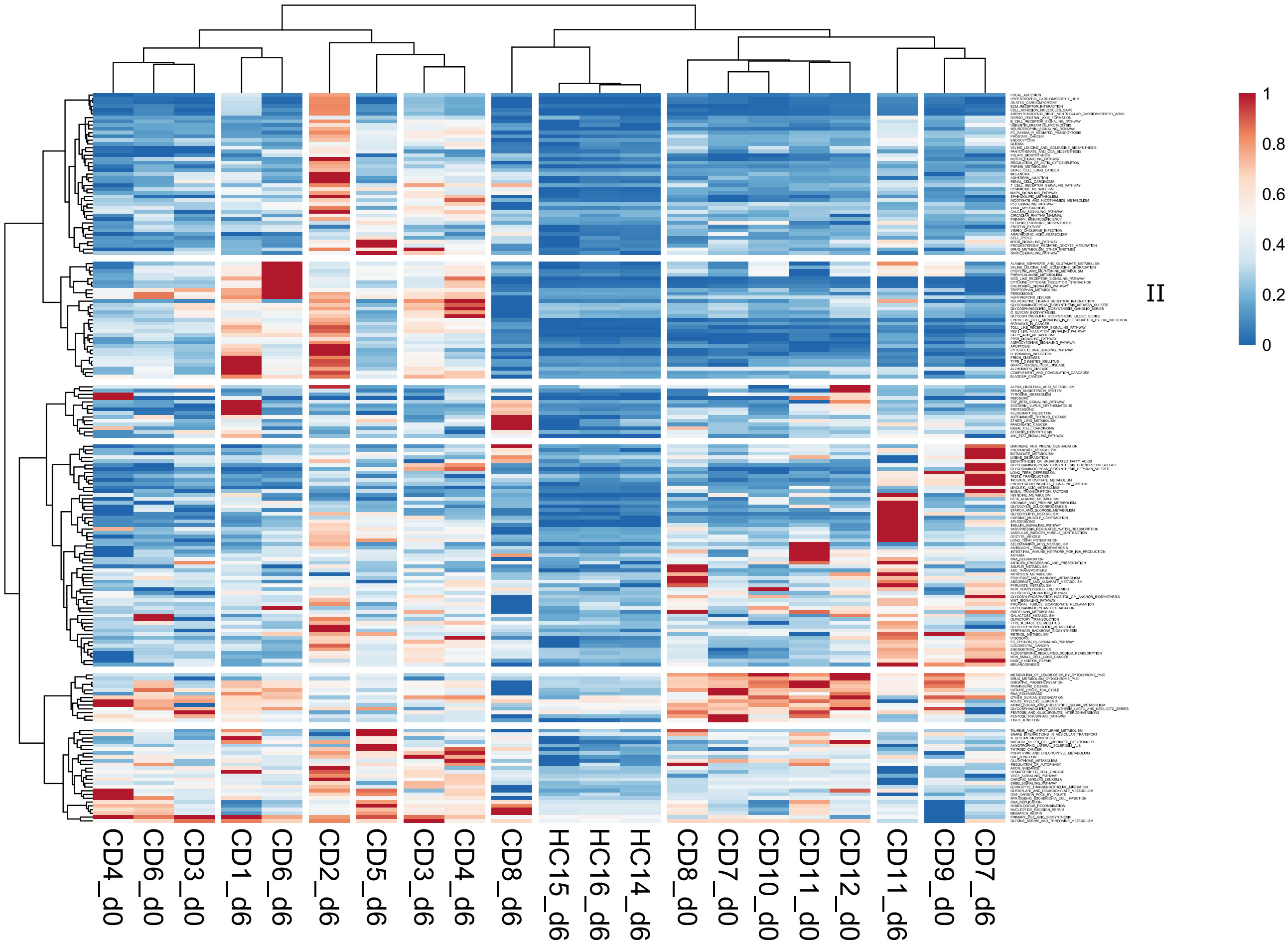

Figure S8: Sample-specific pathway deregulation score heatmap including “exceptional samples”. The distance of each pathway from the “healthy” state is shown using heatmap of all samples. Day 0 samples of patients CD3, 4, and 6 that cluster with CD patient day 6 samples in Figure S1, do the same in this heatmap (column wise clustering of pathway profiles). These day 0 samples have high deregulation in Cluster II of pathways, similar to patient day 6 profiles in this cluster. Cluster II contains immune-response related pathways such as cytokine-cytokine receptor interaction, Toll like receptor signaling and Rig-I like receptor signaling) suggesting that CD3, 4 and 6 had heightened immune activity at day 0 compared to all other patient samples from day 0. On the other hand, day 6 samples from patients CD7, 8 and 11, that were closer to healthy control day 6 samples on Figure S1, have overall pathway profiles similar to patient day 0 samples, especially CD7 and 11, indicating that their pathway profile shows deregulation patterns relatively consistent with CD (with some special exceptions) but not yet as extreme as other day 6 samples.

Table S2: Expression of interferon gamma gene (IFNG) detected in our data. Counts are in transcripts per million extracted from the whole expression dataset. The detection level of IFNG gene was low in our dataset and the gene was not called differentially expressed in all three comparisons, but was predicted to be significantly activated by IPA's upstream regulator prediction method.

	CD7_d0	CD8_d0	CD9_d0	CD10_d0	CD11_d0	CD12_d0	CD1_d6	CD2_d6	CD3_d6	CD4_d6	CD5_d6	CD6_d6	HC15_d6	HC16_d6	HC14_d6		MeanExp
IFNGR1	738	598	645	726	460	581	671	385	590	1076	613	1441	451	591	1028		706
IFNGR2	25	55	36	44	51	17	70	247	231	361	171	96	104	26	42		105
IFNG-AS1	8	0	12	19	7	28	17	0	0	12	0	0	12	0	34		10
IFNG	8	8	0	0	15	23	41	6	0	0	0	0	0	0	0		7
